# The Impact of High‐Fidelity Simulation and Curriculum Integration on Nursing Students’ Competence and Satisfaction: A Quasiexperimental Study

**DOI:** 10.1155/jonm/3985380

**Published:** 2026-03-23

**Authors:** Ahmad Ismail, Hanan Al-Modallal, Njoud Aldardeir, Nada Gomma, Nadia Abd ElHamed Eltohamy

**Affiliations:** ^1^ Nursing Department, Fakeeh College for Medical Sciences, Fakeeh Care Group, Jeddah 21461, Saudi Arabia, fakeehcollege.edu.sa; ^2^ Medical Education Department, Fakeeh College for Medical Sciences, Fakeeh Care Group, Jeddah 21461, Saudi Arabia, fakeehcollege.edu.sa; ^3^ Maternal and Newborn Health Nursing, Faculty of Nursing, Helwan University, Helwan, Egypt, helwan.edu.eg

## Abstract

**Background:**

Curriculum integration and high‐fidelity simulation (HFS) were used separately in nursing education. Little is known about their combined effect on undergraduate nursing students’ competence.

**Aim:**

To assess the impact of HFS and curriculum integration on nursing students’ competence and satisfaction.

**Methods:**

A quasiexperimental study design was employed. Using convenience sampling, two groups of undergraduate nursing students were compared in their management of pregnant women undergoing normal vaginal delivery and immediate newborn care. The intervention group (*n* = 30) received the HFS/curriculum‐integrated session, while the control group (*n* = 30) received the traditional curriculum. The HFS/curriculum‐integrated intervention consisted of five three‐hour sessions, with six students participating in each session. The intervention was conducted in the simulation laboratory of Fakeeh College for Medical Sciences in December 2023 over one week, and study data were collected two weeks after the completion of the educational intervention. Students’ competence was assessed using a structured written examination and an objective structured clinical examination (OSCE), while satisfaction was evaluated using a self‐reported questionnaire. Independent *t*‐tests were used to examine differences in competency between the two groups.

**Results:**

Students who participated in the HFS/curriculum‐integrated sessions demonstrated significantly higher performance in both written knowledge examinations and clinical skills assessments compared to the control group. Knowledge scores were higher for management of normal vaginal delivery (86% vs. 46%) and immediate newborn care (80% vs. 54%), and OSCE scores were also significantly greater for vaginal delivery management (81% vs. 50%) and immediate newborn care (95% vs. 56%) (*p* ≤ 0.05). Students’ satisfaction with the integrated session using HFS was high (91% ± 7.6).

**Conclusion:**

The use of HFS and curriculum integration significantly enhanced nursing students’ competency and satisfaction levels. These findings underscore the value of incorporating HFS and curriculum integration as an effective strategy in nursing education. However, further large‐scale and longitudinal studies are warranted to more comprehensively evaluate the long‐term impact of HFS and curriculum integration on diverse nursing learning outcomes.

**Implication for Nursing Management:**

Nursing management should facilitate the use of HFS and curriculum integration in nursing education, as this approach significantly enhances students’ clinical competence while bridging the gap between theory and practice.

## 1. Introduction

Nursing education is a dynamic and systematic process. Nurse educators can effectively facilitate students’ learning with up‐to‐date trends, evidence‐based practices, and new teaching and learning tools/resources. They aim to facilitate and support students becoming competent, self‐reliant, safe, and compassionate nurses in the clinical setting [1]. Two powerful approaches that are transforming nursing education and significantly enhancing student learning are curriculum integration and simulation [2–5].

Curriculum integration is becoming an increasingly important concept in health professions education. An integrated curriculum refers to breaking down traditional teaching boundaries and incorporating multiple disciplines and perspectives into the curriculum [6]. It combines learning content and courses, which strengthens students’ learning while improving the professional skills of the learners [7, 8]. Within the nursing context, an integrated curriculum brings several benefits to both the learner and the patient. It bridges the gap between theory and practice, enhances patient safety initiatives, and enriches the key competencies needed to be a qualified nurse practitioner [2, 9]. It also facilitates their development as safe, professional, competent, and compassionate healthcare professionals [10]. Nurse educators may provide a curriculum to nurture students’ skills, critical thinking, and knowledge to produce the best‐expected educational outcomes [11, 12].

Simulation is widely used in undergraduate nursing education as a teaching strategy to bridge the gap between theory and clinical practice [13–15]. Simulation in nursing education includes several types that vary in realism and technological complexity. One of them is the high‐fidelity simulation (HFS), which uses computerized manikins that mimic real clinical conditions. It provides students with a safe, controlled environment where they can practice essential clinical skills, decision‐making, and critical thinking without the risk of harming patients [16–20]. Through realistic, scenario‐based learning, students can engage in hands‐on experiences that replicate real‐life clinical situations, enhancing their preparedness for clinical settings. The HFS also promotes active learning, collaboration, satisfaction, empathy, self‐awareness, clinical decision‐making, clinical judgment, and self‐confidence by allowing students to manage complex patient care scenarios, such as emergencies, mental health cases, maternity, pediatric nursing, or airway management [16, 18–24]. Additionally, the debriefing process in HFS encourages reflection, critical analysis, and feedback, helping students improve their performance and understanding [25]. As a result, HFS in undergraduate nursing education enhances both technical and nontechnical skills, contributing to better patient outcomes and a more competent nursing workforce.

Several nursing studies have focused on using HFS or curriculum integration as separate teaching strategies. Although previous studies have demonstrated the effectiveness of HFS or curriculum integration as independent educational strategies, limited research has examined the combined effect of curriculum integration and HFS. Furthermore, the current study uniquely employs a horizontally integrated curriculum that combines women’s health and child health nursing content and evaluates its impact on objective competency outcomes. In this study, student competence refers to the integration of both cognitive and psychomotor abilities required for safe and effective nursing practice. Specifically, competence encompasses theoretical knowledge related to nursing management during normal vaginal delivery and immediate newborn care, as well as clinical assessment and procedural skills necessary to perform these interventions, which were objectively evaluated using a structured knowledge examination and an objective structured clinical examination (OSCE).

## 2. Methods

### 2.1. Design

A quasiexperimental design was employed to compare the levels of competence in nursing management of pregnant women undergoing normal vaginal delivery and immediate newborn care between students exposed to the HFS/curriculum integration and those who were not. A structured written examination and an OSCE assessed students’ competence. Students’ satisfaction with the HFS/curriculum‐integrated sessions was measured using a self‐reported questionnaire.

### 2.2. Setting and Participants

This study occurred at Fakeeh College for Medical Sciences (FCMS). The Bachelor of Nursing program at FCMS is approved by the Ministry of Education of Saudi Arabia and accredited by the National Center for Academic Accreditation and Evaluation (NCAAA). The participants for the current study were third‐year nursing students (2023). Their ages ranged from 21 to 24 years. The first year of the Bachelor of Nursing program is preparatory, focusing on general science, English language, and communication and learning skills courses. Theoretical and clinical nursing courses are offered primarily in the program’s second, third, and fourth years. A full internship year (52 clinical weeks) follows the fourth year, rotating in several departments such as critical care, medical‐surgical, pediatric, maternity, psychiatric, operating room, and emergency [26]. Child health and Women’s health nursing courses are usually offered in the third year of the program after completing the Adult Health Nursing course.

Two groups of students were recruited: those exposed to the HFS/curriculum‐integrated sessions (*n* = 30) and those who were not (*n* = 30). We conducted a post hoc power analysis using Cohen’s d to confirm that the sample size was sufficient. The effect size of the independent *t*‐test is referred to as Cohen’s d [27]. We calculated the effect size based on the mean difference in knowledge test scores between the two groups. We identified an effect size of 1.33, classified as large. According to G∗Power 3 software, with a power of 80%, a significance level of (*p* ≤ 0.05), and an effect size of 1.33, the minimum required sample size is 20 students (10 per group). This means that we recruited an adequate sample. The G∗Power software can calculate the required sample size and power for various statistical methods, such as the independent *t*‐test [28, 29].

### 2.3. Procedure

We have designed an HFS/curriculum‐integrated session between women’s and child health courses using a horizontal model. Horizontal integration is the merging of disciplines within a certain time frame; in this study, the third year. Two topics from each course were integrated: “nursing management during vaginal delivery” in the women’s health nursing and “immediate newborn care” in the child health nursing course. The reason for combining these topics is that both occur simultaneously, reflecting an integration of this particular maternal/newborn stage of life.

The HFS/curriculum‐integrated session’s delivery was conducted in the simulation laboratory at the FCMS by the course instructors. Both instructors have more than 5 years of teaching experience and are adequately trained in the equipment and the HFS manikins. The HFS/curriculum‐integrated session accommodated six students per session. Five three‐hour sessions were conducted. The session was conducted at the end of the second semester when students had already been exposed to the topics separately in each course. A protocol to conduct the integrated session for the two topics was prepared by the faculty educators of the two courses. Data from the study were collected after 2 weeks of the educational interventions. A 2‐week interval was selected based on findings from earlier research indicating that this duration is adequate for evaluating changes after educational interventions [30].

The protocol was conducted in two main parts. The first one was the nursing management during vaginal delivery: presenting a case scenario with patient history and main complaints; gathering all supplies needed for normal vaginal delivery; providers’ assessment of the patient; and performance of care required during vaginal delivery. The second part of the protocol was associated with immediate newborn care. It included the following steps: assembly of essential supplies needed for newborn care, providing immediate care, assessing vital signs, and providing essential measurements for the newborn. We used HFS manikins for birthing women and newborns to conduct the integrated session. A debriefing was conducted after the integrated session to discuss the performance, stimulate reflection, and improve future performance (Table 1).

**TABLE 1:** HFS/curriculum‐integrated session: content, skills, purpose, teaching methods, and duration.

Part	Content/skills	Purpose	Teaching method	Duration (min)
1	Nursing management during vaginal delivery: patient identification and communication, hand hygiene, preparation and use of equipment, maintaining privacy, positioning, key delivery maneuvers (e.g., Ritgen’s maneuver and shoulder delivery), assessment of the patient, and performance of essential delivery care	Enhance students’ competence in safe, evidence‐based vaginal delivery, and application of theoretical knowledge	HFS using birthing manikins, hands‐on practice, scenario‐based learning	90
2	Immediate newborn care: receiving the baby, airway clearance, stimulation, thermal protection, resuscitation when indicated, cord management, administration of vitamin K and eye prophylaxis, newborn identification, APGAR scoring, anthropometric measurements, vital signs assessment, and documentation	Develop competence in essential newborn care, clinical assessment, and rapid decision‐making	HFS using newborn manikins, hands‐on practice, scenario‐based learning	60
3	Debriefing	Reinforce learning outcomes, promote reflection, and improve clinical performance.	Debriefing to discuss performance, stimulate reflection, and enhance future clinical practice	30

### 2.4. Instruments and Outcomes

Two outcomes were assessed: (1) students’ competence regarding nursing care during vaginal delivery and immediate newborn care and (2) students’ satisfaction with the HFS/curriculum‐integrated session. First, students’ competence in nursing care during vaginal delivery and immediate newborn care was measured by two instruments: the knowledge standard examination and procedure skills by OSCE. The knowledge standard examination consisted of 20 multiple‐choice questions developed by the course instructors and validated by a panel of six experts in pediatric and maternity nursing: 10 questions for nursing care during vaginal delivery and 10 for immediate newborn care. The validity of the examination was assessed using the content validity index (CVI), which indicated good content validity (CVI = 0.82). The reliability of the examination was measured using the Kuder–Richardson Formula 20 [31]. It is a test that checks the internal consistency reliability of dichotomous choices when there is a correct answer and one or more wrong answers, like multiple‐choice questions with one true answer [31, 32]. The reliability of the examination was found to be 0.87, indicating good reliability. Procedural skills were assessed by OSCE. The OSCE is a valuable strategy for assessing students’ competence [33].

Items of the OSCE were developed based on the Nursing Lippincott Procedures for nursing care during vaginal delivery and immediate newborn care [34]. Procedural skills assessed through the OSCE included comprehensive care during vaginal delivery and immediate newborn care. For delivery, skills covered patient identification and communication, hand hygiene, preparation and use of equipment, privacy, positioning, and key delivery maneuvers (e.g., Ritgen’s maneuver and shoulder delivery). For newborn care, skills included thermal protection, airway clearance, stimulation, resuscitation when indicated, cord management, administration of vitamin K and eye prophylaxis, newborn identification, APGAR scoring, anthropometric measurements, vital signs assessment, and documentation.

Student satisfaction with the HFS/curriculum‐integrated session survey was assessed using a satisfaction survey. This survey was developed and executed by the Quality Department of the FCMS. The survey was offered in both English and Arabic. The survey consisted of a 14‐question satisfaction questionnaire, scored on a 5‐point agreement scale (1 = *strongly disagree*, and 5 = *strongly agree*). The total satisfaction score is the mean of the 14 items. These 14 items included four items about the role of the facilitator, six items on the effectiveness of the integrated session, and four items on active learning. The satisfaction scale underwent content validation by a panel of six experts in nursing, quality assurance, nursing education, and medical education to ensure clarity, relevance, and alignment with the study objectives. The CVI showed excellent content validity (CVI = 0.94). The internal consistency reliability of this questionnaire using Cronbach’s alpha was 0.89, indicating very good internal consistency.

### 2.5. Data Analysis

The IBM SPSS Statistics software Version 26 was used to analyze the data. The data were analyzed for frequencies, descriptive statistics, and independent *t*‐tests. The independent *t*‐test was used to compare the means of the two groups of students who were exposed to the HFS/curriculum‐integrated session and those who did not have knowledge and competency. We also compared the two groups in their grade point average (GPA) to ensure that the students’ academic level was not a confounding factor that influenced their achievement in the knowledge examination and OSCE. Satisfaction was measured by calculating the mean of the satisfaction survey with the HFS/curriculum‐integrated session of the students.

### 2.6. Ethics

Ethical approval was obtained from the FCMS (454/IRB/2023). Participation in the HFS/curriculum‐integrated session was voluntary. Signed informed consent forms were gathered from the students. The consent form included information about the study, potential benefits, potential harms from the participation, data storage and disposal, and contact information for the ethical review board. Students had the chance to withdraw from the study at any time. Confidentiality of the gathered data was protected by substituting codes for participant identifiers. Data were stored in designated flash storage inside a locked cabinet in the FCMS research storage room. The knowledge examination was run using the Blackboard platform, and skill procedures were assessed using a skills assessment sheet. The satisfaction survey was anonymous and voluntary. A secure platform (SurveyMonkey) was used to collect the data on satisfaction with the HFS/curriculum‐integrated session.

## 3. Results

All participants were female students. Thirty students were exposed to HFS/curriculum‐integrated sessions, and 30 were not. The mean GPA of the HFS/curriculum‐integrated session group was 4.3 ± 0.40, and the GPA of the control group was 4.1 ± 0.70. The two groups had no significant difference in GPA (≥ 0.05). The total knowledge score of the students exposed to the HFS/curriculum‐integrated session was significantly higher than that of the control group (83% vs. 50%, *p* ≤ 0.05).

For the students’ knowledge of nursing care during vaginal delivery, the total knowledge score of the students who were exposed to the HFS/curriculum‐integrated session was significantly higher than the control group’s score (86% vs. 46%, *p* < 0.05). Students exposed to the HFS/curriculum‐integrated session answered all the questions higher than in the control group. The difference in 9 out of 10 questions was significant (*p* < 0.05). The highest mean difference was in the item “The nurse should contact the physician immediately if the patient experiences hypotonic uterine contractions during active labor” (100% vs. 30%) (*p* < 0.05) (Table 2).

**TABLE 2:** Students’ knowledge of nursing care during vaginal delivery.

No	Item	Control group Correct answers mean (%)	HFS/curriculum integration group correct answers mean (%)	*t*	*p* value
1	To reduce the pain during delivery, bilateral pudendal block can be used, mainly targeting the perineal and vaginal areas.	47	100	5.8	0.001
2	The nurse should contact the physician immediately if the patient experiences hypotonic uterine contractions during active labor.	30	100	8.2	0.001
3	The transition phase of labor is the shortest stage, but it is also the most intense for the patient.	57	83	2.3	0.02
4	Internal electronic fetal monitoring can only be used after the patient’s membranes have ruptured and the cervix is dilated to a minimum of 2 cm.	53	70	1.3	0.19
5	Throughout the majority of the first stage of labor, pain is primarily focused in the pelvic region.	50	73	1.88	0.07
6	During prolonged oxytocin infusion, the nurse should closely monitor the patient’s fluid intake and output, as prolonged use can result in severe water intoxication, potentially causing seizures, coma, or even death.	43	70	2.12	0.04
7	The transition phase typically lasts from one to 3 hours.	47	87	3.6	0.001
8	In the second stage of labor, the nurse should evaluate the strength, frequency, and duration of contractions every 15 min.	63	100	4.10	0.001
9	The priority intervention is to prepare for immediate delivery when the patient presents with the following assessment findings: Gravida 2 Para 1, approximately 40 weeks of gestation, contractions occurring every 2 minutes and lasting 45 s, with the fetus at a +4 station in the vertex position.	27	87	5.80	0.001
10	Shifting the client from supine to side‐lying position may quickly resolve variable decelerations in the fetal heart rate, which indicate umbilical cord compression.	47	87	3.60	0.001
Total		46	86	7.6	0.001

*Note: t* = independent *t*‐test.

Abbreviation: HFS = high‐fidelity simulation.

For the students’ knowledge of immediate newborn care, the total knowledge score of students exposed to the HFS/curriculum integrated session was significantly higher than the control group’s mean (80% vs. 54%, *p* < 0.05). Students exposed to the HFS/curriculum integrated session answered 8 out of 10 questions higher than in the control group. The difference in 5 out of 10 questions was significant (*p* < 0.05). The highest mean difference was in the item “To measure the head circumference, the nurse should place the tape measure under the infant’s head, wrap the tape around the occiput, and measure just above the eyebrows so that the largest area of the occiput is included” (87%, vs. 32%) (*p* < 0.05) (Table 3).

**TABLE 3:** Students’ knowledge of immediate newborn care.

No	Item	Control group Correct answers mean (%)	HFS/curriculum integration group correct answers mean (%)	*t*	*p* value
1	To prevent heat loss from evaporation, the nurse wraps the newborn in a warm blanket and thoroughly dries the infant.	67	87	1.85	0.07
2	To measure head circumference, the nurse should wrap the tape measure around the occiput and place it just above the eyebrows to capture the widest part of the occiput.	23	87	6.30	0.001
3	Vitamin K is given to newborns to prevent serious bleeding.	80	87	0.68	0.50
4	The nurse should assess the newborn’s vital signs in a way that minimizes disturbance, measuring respirations first, followed by pulse, and then temperature.	87	87	0	1
5	Keeping the remaining portion of the umbilical cord dry and exposed to air helps prevent infection and promotes faster drying.	57	73	1.35	0.18
6	Vitamin K is given intramuscularly (IM) into the vastus lateralis muscle, which is located on the outer side of the thigh.	90	83	0.75	0.46
7	Eye prophylaxis, such as erythromycin, protects newborn eyes from infections caused by bacteria. The eyes should not be rinsed after applying the medication.	30	87	5.35	0.001
8	The heart rate is the most critical sign assessed in the APGAR score.	43	87	3.88	0.001
9	The respiratory rate in neonates can reach up to 60 breaths per minute and is affected by activity.	53	70	1.33	0.19
10	The newborn’s breathing is abdominal and irregular in both depth and rhythm.	7	57	4.85	0.001
Total		54	80	5.19	0.001

*Note: t* = independent *t*‐test.

Abbreviation: HFS = high‐fidelity simulation.

For the students’ OSCE of nursing care during vaginal delivery, the total score of the students who were exposed to the HFS/curriculum‐integrated session was significantly higher than the control group’s mean (81% vs. 50%, *p* < 0.05). Students exposed to the HFS/curriculum integrated session scored 16 out of the 20 items higher than in the control group. The difference in 15 items was significant (*p* < 0.05). The highest mean difference was in the item “Urge the mother to void or defecate, if able” (90% vs. 32%) (*p* < 0.05) (Table 4).

**TABLE 4:** OSCE results of nursing care during vaginal delivery.

No	Item	Control group Correct answers mean (%)	HFS/curriculum integration group correct answers mean (%)	*t*	*p* value
1	Introduce self to the patient	83	78	0.67	0.51
2	Identify the patient using two identifiers.	79	73	0.57	0.57
3	Perform hand washing and put on PPE, if indicated.	68	69	0.12	0.91
4	Provide patient privacy.	68	64	0.33	0.74
5	Explain the procedure to the patient and gain verbal consent.	68	100	5.12	0.001
6	Prepare the equipment and materials	61	100	6.73	0.001
7	Urge the mother to void or defecate, if able	32	90	9.80	0.001
8	Assist the woman in moving from bed to the delivery table and position on lithotomy.	62	79	1.90	0.06
9	Place padding on the stirrups, lift both legs simultaneously, and make any necessary adjustments.	51	71	2.70	0.009
10	Perform Ritgen’s maneuver when the fetal head crowns the vaginal opening by applying gentle pressure on the woman’s perineum with one hand while the other hand presses on the fetal head. Encourage the mother to blow to help control her pushing effort.	34	87	8.41	0.001
11	Wipe secretions from the infant’s face using a clean, dry gauze, and then use a bulb syringe to suction the mouth and nose.	39	60	2.16	0.04
12	Check for the umbilical cord around the baby’s neck (nuchal cord). If it is loose, gently slip it over the head and shoulders. If it is tight, clamp and cut it before the baby is fully delivered.	41	100	8.31	0.001
13	Following external rotation, the physician gently pulls the fetal head toward the mother’s perineum.	34	87	7.32	0.001
14	Raise the head upward toward the mother’s symphysis pubis.	41	72	4.76	0.001
15	As the head restitutes and rotates, apply a steady, gentle downward traction to help the anterior shoulder pass beneath the symphysis pubis.	46	81	4.95	0.001
16	Gently lift upward to help the posterior shoulder glide over the perineum.	42	77	4.69	0.001
17	Once the shoulders are delivered, the remainder of the infant’s body follows swiftly.	32	81	6.36	0.001
18	Keep the infant in a head‐down position (with the head slightly lower than the body) while using a bulb syringe to suction excess secretions.	33	63	3.66	0.001
19	The infant is typically placed on the mother’s chest to encourage skin‐to‐skin contact.	40	96	7.95	0.001
20	Document and comment on the findings.	36	91	7.80	0.001
Total		50	81	6.7	0.001

*Note: t* = independent *t*‐test.

Abbreviation: HFS = high‐fidelity simulation.

For the students’ OSCE of immediate newborn care, the total score of students exposed to the HFS/curriculum‐integrated session was significantly higher than the control group score (95% vs. 56%, *p* < 0.05). Students exposed to the HFS/curriculum‐integrated sessions scored 20 out of 20 items higher than in the control group. The difference in all of them was significant (*p* < 0.05). The highest mean difference was in the item “Keep the baby’s face down to prevent aspiration” (94% vs. 33%) (*p* < 0.05) (Table 5).

**TABLE 5:** OSCE results of the immediate newborn care.

No	Item	Control group correct answers mean (%)	HFS/curriculum integration group correct answers mean (%)	*t*	*p* value
1	Identify self to the mother.	63	91	3.60	0.001
2	Perform hand hygiene and put on PPE, if indicated.	43	86	6.44	0.001
3	Keep the room warm and adjust the temperature from the radiant warmer to prevent hypothermia.	37	94	8.42	0.001
4	Once the head is delivered, gently wipe the mouth and nose with gauze.	70	100	5.34	0.001
5	Receive the baby in a clean blanket or towel.	60	94	4.88	0.001
6	Keep the baby’s face down to prevent aspiration.	33	94	12.69	0.001
7	Record the time of birth and the baby’s sex, and then announce them clearly so the mother can hear.	69	100	4.73	0.001
8	While drying the baby, stimulate them by gently rubbing up and down along their spine using the heel of your hand.	59	100	10.79	0.001
9	If the baby is not crying or breathing adequately within 30 s after birth, clamp and cut the cord, and then start resuscitation. If the baby is breathing normally, proceed with the remaining essential newborn care steps.	51	100	11.79	0.001
10	Put the newborn under a radiant warmer in a side‐lying or Trendelenburg position to prevent hyperthermia.	47	100	8.45	0.001
11	Dry the baby thoroughly and remove the wet linen. Keep the baby warm.	53	100	13.61	0.001
12	Perform suction of the nasopharynx and oropharynx.	48	100	8.00	0.001
13	Clamp and cut the cord with an aseptic solution.	57	100	11.95	0.001
14	Give eye care (while the mother holds the baby). Put erythromycin eye ointment into the eyes of the newborn baby shortly after breastfeeding and within 1 hour of being born.	79	100	4.83	0.001
15	Administer Inj. Vitamin K (IM).	69	100	5.89	0.001
16	Secure two identification bands on the baby’s wrist and ankle, including the mother’s and, if available, the father’s names, the baby’s sex, and the date and time of birth.	64	96	4.63	0.001
17	Evaluate the newborn’s heart rate, respiration, muscle tone, reflex irritability, and color initially at 1 minute (APGAR score five categories). Calculate and sum the scores from each category to get the final APGAR score.	63	100	9.92	0.001
18	Take the anthropometric measurements: weight, length, head circumference, chest circumference, and abdominal girth.	60	76	3.60	0.001
19	Assess the vital signs by measuring the respiratory rate, heart rate, temperature, and blood pressure.	62	100	12.23	0.001
20	Document and comment on the findings.	37	81	6.74	0.001
Total		56	95	20.77	0.001

*Note: t* = independent t‐test.

Abbreviation: HFS = high‐fidelity simulation.

The total students’ satisfaction with the HFS/curriculum‐integrated session was high (91% ± 7.6). Students’ satisfaction with the role of the instructor was high (92% ± 7.48). The highest satisfaction score in this domain was “The instructor facilitated the integrated teaching sessions well” (95% ± 9). Students’ satisfaction with the effectiveness of the integrated session was also high (90% ± 7.78). The highest satisfaction score in the effectiveness of the integrated session domain was “The integrated teaching sessions provided me with a better understanding of topics” and “Enough time was devoted to conducting the integrated teaching sessions” (95% ± 9). Students’ satisfaction with active learning was also high (91% ± 9.64). The highest satisfaction score in this domain was in the item “Integrated teaching sessions are more effective than didactic lectures” (95% ± 9) (Table 6).

**TABLE 6:** Students’ satisfaction with the HFS/curriculum‐integrated session.

No	Item	Mean (%)	SD
*Role of the instructor*		*92*	*7.48*
1	The instructor encourages my participation.	94	9.32
2	The instructor paid enough personal attention and support to the students.	88	9.96
3	The instructors promoted active interaction between themselves and the students.	91	15.52
4	The instructor facilitated the integrated teaching sessions well.	95	9.00

*The effectiveness of the session*		*90*	*7.78*
5	The integrated teaching sessions increased my interest in the discussed topics.	88	9.96
6	The integrated teaching sessions gave me knowledge and skills that are helpful in clinical practice.	82	13.24
7	The integrated teaching sessions provided me with better understanding of topics.	95	9.00
8	The topics discussed in integrated sessions were relevant with the intended learning outcomes (ILOs).	91	10.08
9	The integrated teaching sessions were well organized.	91	15.52
10	Enough time was devoted for conducting the integrated teaching sessions.	95	9.00

*Active learning*		*91*	*9.64*
11	The integrated teaching sessions enhanced the interaction between instructor and student.	94	9.32
12	The integrated teaching sessions facilitated collaborative learning among students.	88	15.40
13	Integrated teaching sessions are more effective compared to traditional didactic lectures.	95	9.00
14	The number of students in integrated teaching sessions was appropriate to encourage active student participation.	89	17.96
Total		91	7.6

Abbreviation: SD = standard deviation.

## 4. Discussion

The findings of this study demonstrate that the HFS and curriculum integration strategy significantly improves nursing students’ competence and satisfaction. Students exposed to the HFS/curriculum‐integrated sessions achieved markedly higher scores in written knowledge assessments and OSCEs across both vaginal delivery management and immediate newborn care. These findings are consistent with a growing body of evidence supporting the effectiveness of simulation‐based education in enhancing both cognitive and psychomotor competencies among nursing students [13, 16–23]. Further, high levels of satisfaction concerning the role of the instructor, the effectiveness of the integration session, and active learning were reported by the HFS/curriculum integration group. The lack of a significant difference in GPA between the two groups reinforces that the superior performance of the HFS/curriculum integration group is attributable to the educational intervention rather than baseline academic ability.

In the current study, horizontal integration of connecting themes and ideas for two courses (child health and women’s health) offered to students of the same level at the school was implemented. As a result of integration, students’ knowledge and skills were significantly improved. Consistent with this finding, similar studies pointed to an improvement in students’ final examinations by 14%–16% [35]. Further, a noticeable improvement in students’ GPA [7] was identified, making it possible to produce better graduates. The long‐term effects of curriculum integration go beyond graduation, where there is evidence of improved students’ performance at nationally set examinations [36]. Such findings can be explained by students in the integration group demonstrating active learning. They were involved in caring for a birthing mother and a newborn simultaneously during the integration. This practice enhanced students’ critical judgment and clinical reasoning skills [7], depending on the unexpected upcoming situation of the birthing manikin and the newborn manikin. Further, students’ exposure to an unknown situation of the birthing mother and the baby formulated a problem‐based educational situation, which further enhanced students’ cognitive and clinical reasoning skills [37].

The improvement in knowledge scores, particularly in critical items such as immediate recognition of abnormal labor patterns and accurate newborn assessment techniques, suggests that HFS provides a realistic, immersive environment that enhances clinical reasoning. This aligns with studies reporting that HFS facilitates deeper understanding by allowing learners to actively engage in decision‐making and problem‐solving [22, 23, 38]. Moreover, the fact that 9 out of 10 questions in the vaginal delivery knowledge test and 8 out of 10 in newborn care were better answered by the HFS/curriculum integration group (majority with statistically significant differences) illustrates the comprehensive impact of HFS on learning retention. Similarly, OSCE performance saw dramatic improvements in the HFS/curriculum integration group, especially in complex tasks such as performing Ritgen’s maneuver and ensuring neonatal resuscitation readiness. The ability of students to perform better in real‐time, skill‐based assessments after HFS/curriculum integration exposure confirms its role in bridging the gap between theory and practice. These findings resonate with previous research indicating that HFS leads to improved clinical judgment, increased procedural confidence, and better psychomotor skills [23, 39, 40].

In this study, nursing students were given a safe and controlled environment to practice essential clinical skills, make decisions, and apply critical thinking without any risk to real patients. They were engaged in a realistic, scenario‐based simulation that mirrors real clinical environments. These simulations support active learning and foster teamwork and confidence by exposing those students to complex care situations, including emergencies and specialized fields like maternity or pediatric nursing [24]. Furthermore, the debriefing sessions following simulations promote self‐reflection, critical thinking, and constructive feedback, which help students refine their skills and knowledge (Neill & Wotton, 2011). In this study, students benefited from the two educational strategies (curriculum integration and HFS). This boosted the level of acquired knowledge and skills regarding the care of women undergoing vaginal delivery and immediate newborn care.

Students in the HFS/curriculum integration group had the opportunity to connect theory and clinical practice. This connection helped them build a general picture of the woman’s health problem as well as the baby’s, including their physiological measures and their bodies’ responses to such measures. This general picture enables the students to act accordingly in managing the birthing situation and caring for the newborn, as they can easily retrieve what they had learned in the theory part. The role of the faculty members in bridging the gap between theory and practice in integrated classes is of high importance [9], and this was evidently present during the integration session.

The high satisfaction levels among students, particularly regarding instructor engagement, session organization, and opportunities for active learning, highlight the positive perception of HFS/curriculum integration. The rating of 91% for overall satisfaction and 95% for the effectiveness of the sessions affirms that HFS/curriculum integration not only supports learning but also fosters a positive educational experience. This is critical, as learner satisfaction is a significant factor in motivation, engagement, and academic success in healthcare education [41]. One of the factors assumed to contribute to students’ satisfaction is that the integration process enhances students’ attendance in classes. More class attendance means more understanding of the content material and more collaboration. One study supported this idea, where students’ attendance in integration classes and collaboration within the class increased by 60%–65% [42].

Another factor contributing to students’ satisfaction with HFS/curriculum integration is students’ awareness of the construction of the integration situation. Students in the simulation laboratory were expected to face a birthing mother with specific health conditions that they already had an idea about during the theory sessions. Further, they also expected to face a newborn with certain health conditions that they had already studied. Students were prepared to think and implement proper actions accordingly based on the conceptual information and psychomotor skills that they had already learned. Earlier literature supports this idea by stressing that curriculum integration increases a wide range of cognitive skills in students [36]. Proper and prompt actions taken for the mother and baby during the integration session represented the “active learning” part of the student’s satisfaction.

The availability of the course instructor during the HFS/integration session is essential. The instructor is the mediator between the course material and students. He/she directs students’ actions by clarifying current situations. In other words, the instructor plays the role of an interpreter who translates the theory content into learning experiences. Course instructors can bring their knowledge and expertise in a certain area to students to shape the clinical situation and guide students’ actions in that situation. Thereafter, the students in the current study were satisfied with the course instructor, as the latter encouraged student participation by showing attention and support to the students. Words of encouragement and compliments on students’ proper actions also explain students’ satisfaction with the instructors’ part during the integration sessions.

Curriculum integration in nursing education is a powerful approach that aims to ensure that students acquire a comprehensive understanding of various healthcare concepts by integrating multiple disciplines into their learning experience. By combining content from different areas, students can develop a well‐rounded perspective that better prepares them for the complexities of real‐world nursing practice. This integrated approach helps students see the interconnectedness of different facets of healthcare and fosters critical‐thinking skills necessary for making informed decisions in patient care [42–44].

The HFS is an effective tool for improving nursing students’ clinical skills, critical thinking, and decision‐making abilities because it immerses students in realistic, controlled clinical scenarios that closely replicate real‐life patient care situations. This hands‐on experience allows students to practice and refine essential clinical tasks without the risk of harming patients. It also promotes critical thinking by presenting complex, dynamic scenarios that require students to analyze information, recognize problems, and respond appropriately under pressure. As students navigate these challenging situations, they develop problem‐solving skills that are essential for effective clinical judgment. Students are also placed in situations that require quick and informed choices based on clinical evidence and patient assessment [13–15, 17, 45]. The current study provides evidence for the effectiveness of HFS/curriculum integration between two courses (women’s health and child health). Using HFS with curriculum integration brought the benefits of both strategies. This strategy may be adopted as a valuable and effective teaching strategy in courses with clinical or laboratory parts.

## 5. Limitations

Some limitations of this experimental study should be considered. First, this study assessed the HFS/curriculum integration between two subjects (nursing management of women undergoing vaginal delivery and immediate newborn care). To get a more accurate picture, there is a need to assess the impact of curriculum integration using other subjects. Second, this study used a small and convenient sample from one setting, which may affect the generalization beyond this setting. While the large effect size suggests a meaningful educational impact of the HFS‐integrated curriculum, findings should be interpreted carefully and may not be directly generalizable to other nursing programs or contexts. Third, this study did not capture the instructors’ experience of curriculum integration. The experience and suggestions of the instructors are very important to capture to get a more comprehensive picture of curriculum integration. Although the outcomes are highly encouraging, the results also suggest the need for broader implementation and further investigation. Future studies could explore the long‐term retention of skills acquired through HFS and curriculum integration, its cost‐effectiveness, and its impact across different areas of nursing education/care and student populations. In addition, qualitative feedback from students and instructors could offer deeper insights into the mechanisms by which simulation enhances learning. Future multi‐institutional studies with larger and more diverse samples are needed to validate and extend the findings of the current study.

## 6. Conclusion

Nursing students exposed to HFS/curriculum‐integrated sessions between the child and women’s health nursing courses achieved higher scores in knowledge and skills than those who did not (*p* ≤ 0.05). The satisfaction score with the HFS/curriculum‐integrated session was high, as perceived by the students who were exposed to the HFS/curriculum‐integrated session. This study is among the few to empirically examine the combined use of curriculum integration and HFS, rather than evaluating these educational strategies in isolation. Nurse educators may consider using the curriculum integration and HFS strategy in nursing undergraduate education. More multi‐institutional studies with larger and more diverse samples are needed to further evaluate the impact of curriculum integration using HFS in nursing education.

## 7. Implications for Nursing Management

The findings of the current study highlight that nursing management at educational institutions or nursing education accreditation and regulatory agencies should strategically facilitate and encourage the use of the HFS and curriculum integration strategy to enhance students’ cognitive and psychomotor competencies while bridging theory and practice. This requires institutional support through investment in HFS infrastructure, structured faculty development, and competency‐based assessment frameworks. Additionally, HFS and curriculum integration should be embedded within quality assurance and policy frameworks to promote patient safety, workforce readiness, and continuous program improvement.

## Author Contributions

Ahmad Ismail: conceptualization, data curation, formal analysis, investigation, methodology, project administration, software, visualization, writing–original draft, and writing–review and editing; Hanan Al‐Modallal: conceptualization, formal analysis, investigation, methodology, project administration, validation, visualization, writing–original draft, and writing–review and editing; Njoud Aldardeir: conceptualization, investigation, methodology, supervision, validation, visualization, writing–original draft, and writing–review and editing; Nadia Abd ElHamed Eltohamy: conceptualization, investigation, methodology, project administration, validation, visualization, writing–original draft, and writing–review and editing; Nada Gomma: conceptualization, data curation, investigation, methodology, project administration, resources, validation, visualization, and writing–original draft.

## Funding

No funding was received for this manuscript.

## Conflicts of Interest

The authors declare no conflicts of interest.

## Data Availability

The data that support the findings of this study are available upon request from the corresponding author. The data are not publicly available due to privacy or ethical restrictions.
